# Arsenic Removal by Liquid Membranes

**DOI:** 10.3390/membranes5020150

**Published:** 2015-03-27

**Authors:** Tiziana Marino, Alberto Figoli

**Affiliations:** Institute on Membrane Technology, ITM-CNR, Via Pietro Bucci 17/c, 87030, Rende (CS), Italy; E-Mail: t.marino@itm.cnr.it

**Keywords:** arsenic removal, liquid membranes, supported liquid membranes, emulsion liquid membranes

## Abstract

Water contamination with harmful arsenic compounds represents one of the most serious calamities of the last two centuries. Natural occurrence of the toxic metal has been revealed recently for 21 countries worldwide; the risk of arsenic intoxication is particularly high in Bangladesh and India but recently also Europe is facing similar problem. Liquid membranes (LMs) look like a promising alternative to the existing removal processes, showing numerous advantages in terms of energy consumption, efficiency, selectivity, and operational costs. The development of different LM configurations has been a matter of investigation by several researching groups, especially for the removal of As(III) and As(V) from aqueous solutions. Most of these LM systems are based on the use of phosphine oxides as carriers, when the metal removal is from sulfuric acid media. Particularly promising for water treatment is the hollow fiber supported liquid membrane (HFSLM) configuration, which offers high selectivity, easy transport of the targeted metal ions, large surface area, and non-stop flow process. The choice of organic extractant(s) plays an essential role in the efficiency of the arsenic removal. Emulsion liquid membrane (ELM) systems have not been extensively investigated so far, although encouraging results have started to appear in the literature. For such LM configuration, the most relevant step toward efficiency is the choice of the surfactant type and its concentration.

## 1. Introduction

**Figure 1:**
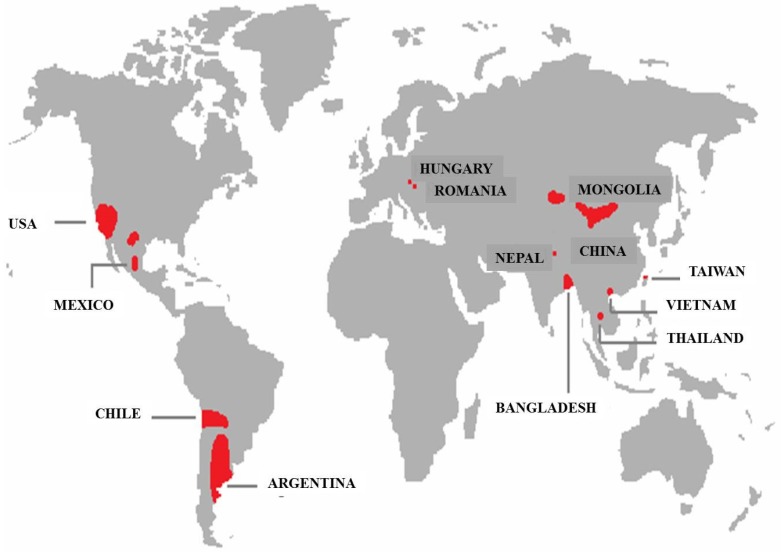
Global arsenic occurrence in groundwater.

Arsenic (As) is a toxic metal that derives its name from the Greek word “arsenikon” meaning “yellow orpiment” [1]. The metalloid element occurs in the nature in various oxidation states, such as As(V), As(III), As(0) and As (-III). Environmental species include arsenious acids (H_3_AsO_3_, H_3_AsO_3_, H_3_AsO_3_^2−^), arsenic acids (H_3_AsO_4_, H_3_AsO^4–^, H_3_AsO_4_^2−^), arsenites (AsO_3_^3−^, As(OH)_3_, As(OH)_4_^−^, AsO_2_OH_2_^−^), arsenates (AsO_4_^3−^, HAsO_4_^2−^, H_2_AsO_4_^−^), methylarsenic acid (CH_5_AsO_3_), dimethylarsinic acid (C_2_H_7_AsO_2_), arsine (AsH_3_), *etc.* The hazardous effect is strongly related to its oxidation state [2]. The two predominant species—arsenite and arsenate, in which the oxidation states are As(III) and As(V)—have become problematic in many areas in the world because of their high toxicity for humans and the environment [3,4]. In particular, arsenite, although it is present at low concentrations in water, is more toxic than the other species. Serious pollution has been registered recently in the USA, Canada, Mexico, Chile, Argentina, Hungary, Poland, China, Bangladesh, New Zeland, Japan, India, and Italy [5,6,7,8,9,10] (Figure 1). Bangladesh and India are the two countries with the highest contamination risk.

It has been demonstrated that the inorganic form is a hazardous human carcinogen and mutagen, which increases the risk for cancer of the kidney, liver, skin, lungs, and bladder, as well as spontaneous abortion and neonatal death [11]. The 1993 edition of the World Health Organization (WHO) guidelines fixed at 0.01 mg/L the provisional guideline content in drinking water, which was accepted by many bodies, including the European Union and the Environmental Protection Agency (EPA) of the USA, as the standard value [12]. However, in other countries the value approved in the earlier edition (0.05 mg/L) was maintained temporarily as standard before working on the arsenic contamination problem [13].

Considering its highly toxic effects, the presence of arsenic in water supplies represents a serious problem for the public health, and a challenge for the world’s researchers.

The presence of arsenic in natural groundwater is mainly associated to the process of leaching from the source rocks and volcanic deposits that contain the metalloid. There exist more than 300 ores containing arsenic as a primary constituent such as mineral enargite and many others in which it is contained in trace amounts. However, man-made pollutants deriving from the combustion of fossil fuels or the use of materials in agricultural manufacture also lead to high arsenic levels in water and groundwater [14]. Inorganic arsenic was widely applied as an herbicide and wood preservative in the 1980s and 1990s. Lead arsenate represented the primary insecticide for fruit orchards before the advent of dichlorodiphenyltrichloroethane (DDT) in 1947 [15]. Furthermore, arsenicals have been used in the manufacture of glass, desiccants, alloys, and electronics.

Arsenic contamination has become a more and more serious issue and tackling it is an important task for ensuring healthy living in the world. The development of new technologies able to remove arsenic from contaminated water represents a challenge for the researchers and can open up new possibilities for the uncontaminated water recovery. Traditional methodologies for metal removal involve adsorption, oxidation/precipitation, and coagulation/coprecipitation [16,17,18].

Among them, the adsorption procedure [19,20,21], mainly based on the use of activated carbons, is particularly efficient for the removal of both As(III) and As(V) species and offers the possibility to use a simple apparatus. However, it presents high costs, which impedes its use on a large scale, as well as high toxic solid waste production and the need for periodic replacement of the specific As resin.

The oxidation/precipitation procedure is another simple, low-cost method that also contributes to the oxidation of other organic/inorganic compounds present in water. The main drawback is the need for meticulous pH control and an oxidation step.

Coagulation/co-precipitation offers the advantages of effectiveness over a wide range of pH and simplicity of operation, but it leads to the production of toxic sludge and several operational steps such as pre-oxidation, sedimentation, and filtration.

Membrane processes represent a promising alternative for arsenic removal, offering the possibility to carry out the separation continuously in one step, under mild operational conditions and with sustainable costs [22,23,24,25,26]. They offer highly efficient As removal without producing toxic solid waste.

One of the emerging membrane technologies for As removal is represented by membrane contactors, even if up to now only few studies have been reported in this field. They are based on the use of microporous hydrophobic membranes, which act as barriers between phases thus allowing the one-step removal of water vapor and volatile compounds from aqueous solutions, without any pre-oxidation step [27,28,29].

In recent years, increasing attention has been paid to another membrane process, the liquid membrane (LM) [30,31,32,33,34]. This is based on the use of liquids impregnated in the pores of membranes; it is not miscible with the feed and product phases. LMs can be efficiently used for wastewater treatment, biotechnology applications, and chemical/biomedical engineering systems. They offer the possibility to operate highly selective separation and recovery of chemical compounds from dilute aqueous solutions, leading to extraction and subsequent stripping operations in a single step [35]. Furthermore, LMs systems allow us to obtain high permeability, especially compared to solid membranes, due to higher diffusion coefficients in liquids, low operating costs, and easy feasibility [36,37,38].

## 2. Liquid Membranes

A LM is generally composed of a thin layer (membrane) containing an organic phase and separating two aqueous solutions. Also, the reverse configuration can be applied. The transport is based on liquid–liquid extraction and continuously operated membrane systems. A polymeric/inorganic microporous membrane can be used as a carrier, as a barrier, or not used. The mass transfer process through the LM basically consists of the solution-diffusion mechanism. The separation involves the solute diffusion through the membrane, due to a chemical/electrical gradient as driving force. However, the efficiency and selectivity of the separation process can be improved by using specific carriers or chemical compounds or applying electrical impulses.

**Figure 2:**
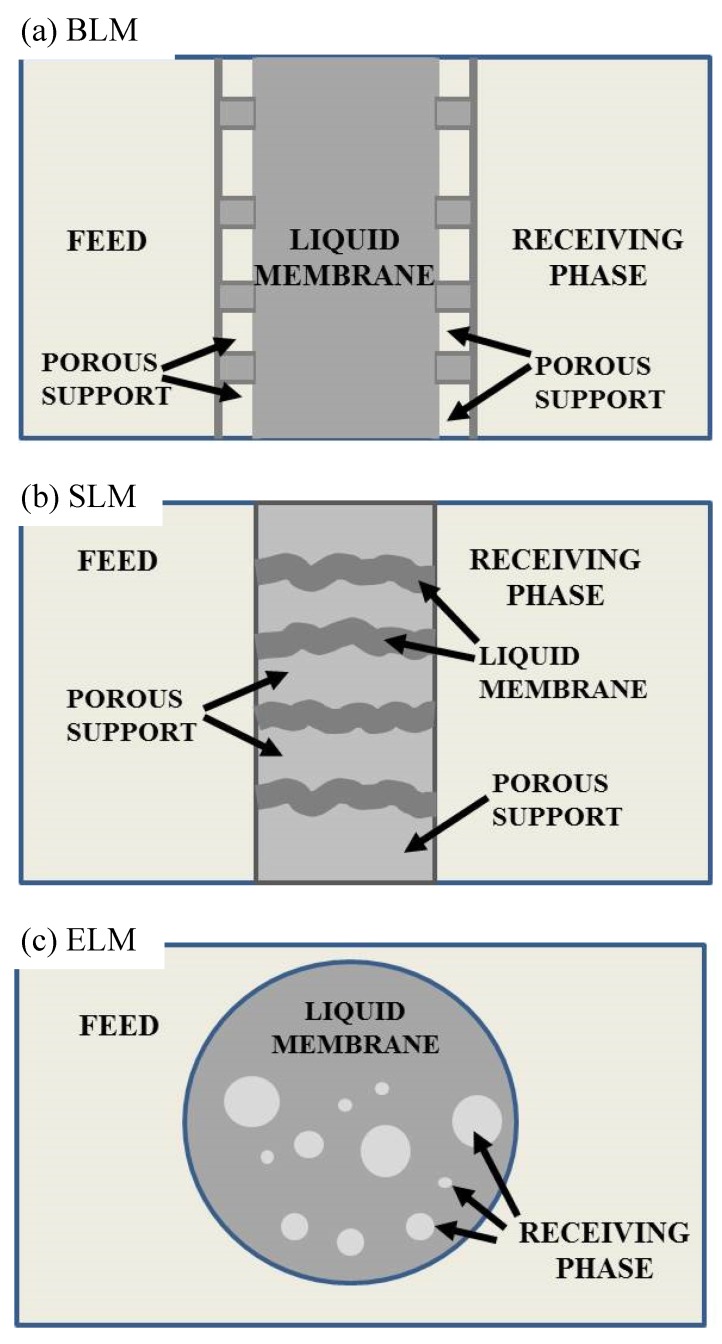
Three configuration models of LMs.

According to the configuration model, LM processes can be classified into three types: bulk liquid membrane (BLM), emulsion liquid membrane (ELM), and supported or immobilized liquid membrane (SLM) (Figure 2) [30,31].

BLM consists of an aqueous (feed) and acceptor phases separated by a bulk organic liquid one. BLMs are based on a simple design for carrying out liquid membrane operations, but present at the same time some disadvantages, such as small membrane surface, which limits its application at an industrial scale [39].

SLMs are based on the use of a porous, polymeric, or ceramic membrane that supports the organic phase and divides the donor phase and the receptor aqueous solution. By capillarity, the organic or carrier phase completely fills the pores of the solid membrane, leading to a relatively stable and heterogeneous solid–liquid membrane. This configuration is characterized by a high mechanical resistance, due to the presence of the solid support. Furthermore, when a solid membrane is hydrophobic, it promotes wetting by the organic solution and the rejection of the aqueous phases. The SLM operational systems commonly used are thin flat-sheet supports, hollow fibers, and spiral wound modules [40].

ELMs are made of an aqueous, internal stripping phase stabilized by oil soluble surfactants and dispersed droplets with very small dimension (1–10 µm) inside an oil phase. The resulting water/oil emulsion is further dispersed as globules of about 0.1–2 mm in another aqueous solution, which represents the donor phase. This type of configuration offers a larger surface area, improving the transport rate through the membrane.

### Liquid Membranes for Arsenic Removal

**Figure 3:**
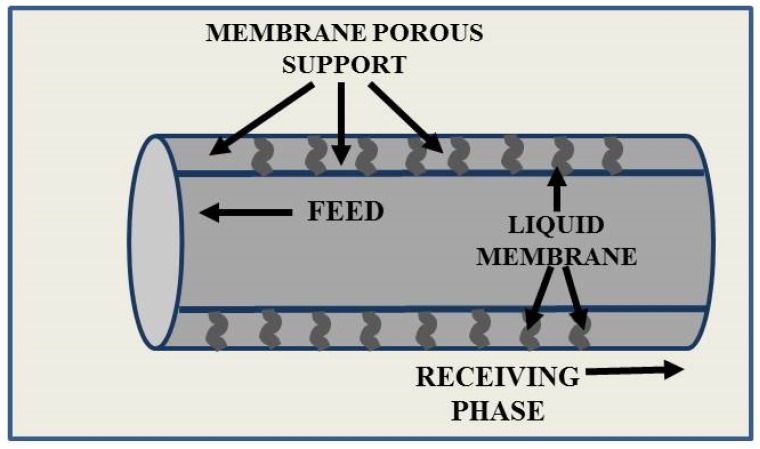
Hollow fiber supported liquid membrane system.

Liquid membranes allow us to combine arsenic ion extraction and stripping processes, which are generally performed in two separate phases in conventional processes such as solvent extraction, into a single step. A one-step process offers the maximum driving force for the removal of toxic chemical species, leading to better clean-up and higher arsenic recovery. The development of LMs for this harmful compound has been studied by several authors [41,42,43]. Particular attention has been devoted to the use of a hydrophobic microporous hollow fiber supported liquid membrane (HFSLM), in which organic extractant molecules are embedded in the membrane pores, allowing the diffusion of the target component through the membrane and preventing at the same time the diffusion of other components. A typical HFSLM consists of three phases: feed, liquid membrane, and stripping solution (Figure 3). Generally, the feed contains the target component, which is transferred during the diffusion through the liquid membrane, in the stripping phase. This LM configuration, combining a solvent extraction and the mass transfer across the membrane, offers several advantages for arsenic removal, such as large surface area to volume ratio and continuous flow operation.

Hylton *et al.* [44] proposed the fabrication of microfluidic membrane extraction systems, which incorporate hollow fiber membranes for the continuous micro-scale extraction of arsenic. Polycarbonate (PC) and polymethylmethacylate (PMMA) were studied as potential materials for device assembly. The first one was able to absorb metals such as copper and arsenic and react with the organic solvent immobilized in the membrane pores, leading to discoloration and disintegration. On the contrary, PMMA did not absorb the metal and demonstrated high mechanical resistance for more than 10 extraction tests. Modules with different dimensions were investigated and dibutyl butylphosphonate (DBBP) and tributyl phosphate (TBP) were used as arsenic extractants [45,46,47]. DBBP created with arsenic a reversible complex at low pH, which was driven across the acceptor, re-ionized using high pH values, and finally concentrated. As solid membrane a commercial polypropylene (PP) hollow fiber was used. The selective organic liquid (DBBP/TBP mixture) was fixed within the pores of the hydrophobic membrane to form the liquid membrane. The membrane module led to donor flow rates of about 1.5 mL min^−1^, with a linear extraction in the time and allowing the ppb level detection by colorimetry tests, which could potentially be lowered using a more sensitive technique. This approach could lead to the development of cost-effective real-time monitoring devices for arsenic detection in water.

Sangtumrong [47] obtained the concomitant separation of Hg(II) and As(III) ions from chloride media using an HFSLM system. The presence of both toxic metals is a relevant environmental pollution problem especially for oil and gas deposits in the Gulf of Thailand [48]. Hg(II) and As(III) compounds are supposed to originate in petroleum, carbonaceous shale, and tin stones in/near reservoirs, leading, consequently, to contaminated water [49]. In view of the risk to which the populations of the interested areas are exposed, the separation of arsenic and mercury ions from water represents one of the major interests in the hydrometallurgical industry. Since the commonly used treatment processes such as adsorption, precipitation, and ion exchange [16,17,18] aim to achieve the complete abatement of the metal species, LMs offer the possibility to separate and recovery the chemical compounds, with a significant advantage for the economy and environment protection. In the work of Sangtumrong *et al.* [47], pure mercury ions were efficiently separated from arsenic ones by using tri-n-octylamine triocthyamine (TOA) dissolved in toluene as an extractant compound. NaOH was used in the stripping solution and HCl in the feed phase. Commercial microporous polypropylene fibers were used as support in the HFSLM system. Since in the chloride media Hg(II) formed anion complexes, they could be driven through the membrane by a concentration gradient of hydrogen ions, which moved in the same direction as the mercury ions. In contrast, As(III) could not react with TOA molecules and thus remained in the raffinate. Experiments on the influence of NaOH concentration on recovery efficiency were carried out, leading to optimal results when 0.4 M was used. Similar tests on TOA and HCl content showed the best mercury percentage extraction and recovery were 2 vol %. and 0.2 M, respectively. The study presented by Sangtumrong *et al.* [47] offered a relevant contribution to the recovery of harmful arsenic and mercury metals, especially in areas of the Gulf of Thailand where the presence of toxic metals represent an extremely dangerous risk to many life forms.

Lothongkum *et al.* [48] operated the simultaneous separation of arsenic and mercury ions from natural gas coproduced using a HFSLM system. The effect of several extractant compounds such as Aliquat 336, Cyanex 923, and Cyanex 471 (tri-isobutylphosphine sulphide) dissolved in toluene was investigated. The influence of sulfuric acid—chosen as a co-extractant—concentration in the feed solution as well as that of the stripping solution (NaOH, distilled water, HNO_3_, H_2_SO_4_, and thiourea) was also studied. The obtained data showed that it was possible to separate arsenic and mercury, although a better removal performance was obtained for mercury ions. Among the extractant mixtures used**,** 0.06 M Cyanex 471, 0.22 M Aliquat 336, and 0.1 M thiourea as the stripping solution with 0.2 M H_2_SO_4_ in feed solution showed a synergic effect on arsenic and mercury extraction. An efficient arsenic removal of 94% was obtained after three cycles of separation processes, which led to a metal content reduction below 250 ppb.

**Figure 4:**
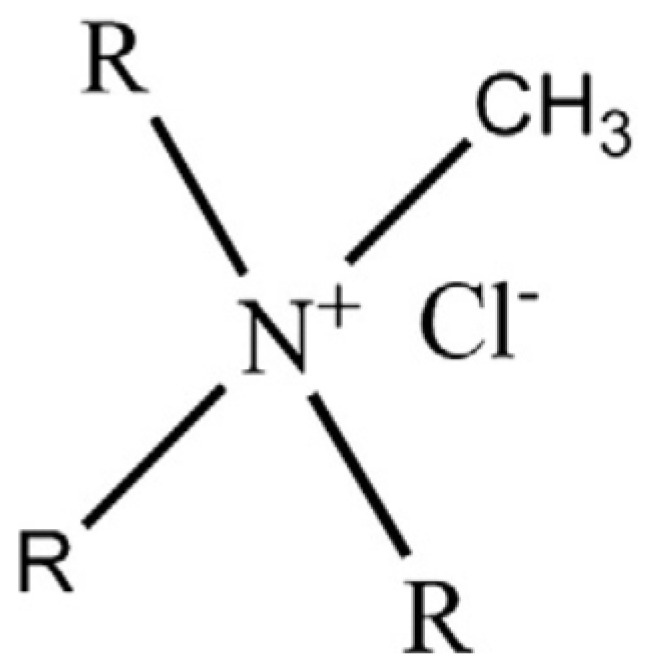
Methyltrioctylammonium chloride (Aliquat 336).

In another work, Pancharoen *et al.* [49] efficiently separated arsenic from produced water with simultaneous gas separation in the Gulf of Thailand using a HFSLM system. Different compounds, such as Cyanex 923, TBP, bis(2, 4, 4-trimethylpentyl) dithiophosphinic acid (Cyanex 301), TOA, and methyltrioctylammonium chloride (Aliquat 336, Figure 4) were tested as extractants, while sodium hydroxide was chosen as the stripping solution.

**Figure 5:**
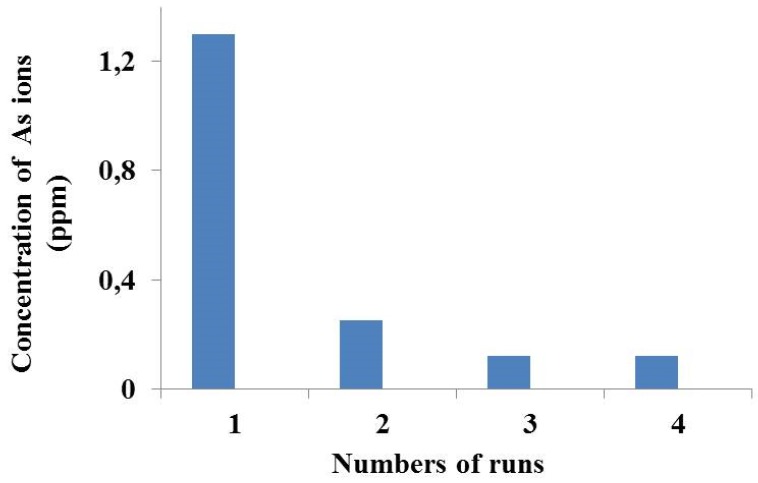
Ppm of arsenic ions from outlet feed solution against the number of cycle operations through HFSLM using Aliquat 336 35% (v/v) and 0.5M NaOH. Feed flow rate: 100 mL/min; Stripping solution flow rate: 100 mL/min [49].

High percentages of arsenic ion recovery were observed when Aliquat 336 was employed, due to the extractant’s ability to react with the dissociated (H_2_AsO_4_^−^ and HAsO_4_) and undissociated (H_3_AsO_3_) metal species. The best results were obtained using the HFSLM containing 0.5 M stripping solution and 0.75 M carrier. Working under these operational conditions, after several separation runs through the HFSLM, it was possible to successfully reduce As ion content in the produced water from the gas separation plant in the Gulf of Thailand at levels lower than 250 parts per billion (ppb), which represents the permissible limit established by the Ministry of Industry in Thailand [50] (Figure 5).

The other organic compounds, Cyanex 923, TBP, and TOA, led to low extractability and several drawbacks, such as the formation of extractant–arsenic ion complexes and the ability to react only with dissociated or undissociated arsenic compounds. The research conducted by Pancharoen *et al.* [49] proved that the molecular type of organic carrier plays a key role in the efficiency of the SLM system.

A further investigation was conducted on the influence of the number of runs through the hollow fiber membrane module on arsenic ion extraction and recovery. The obtained results showed that, at 150 mins (third cycle operation), the HFSLM led to an arsenic extraction of 91% and a recovery of 72% (using 35% v/v of Aliquat 336 and a 0.5 M NaOH solution).

As(III) and As(V) ions were efficiently separated from a sulfate media by Prapasawat [51] using Cyanex 923 (a mixture of phosphine oxide) dissolved in toluene with water as the stripping solution and using HFSLMs. Experimental data showed that more As(V) could be extracted than As(III).

**Figure 6:**
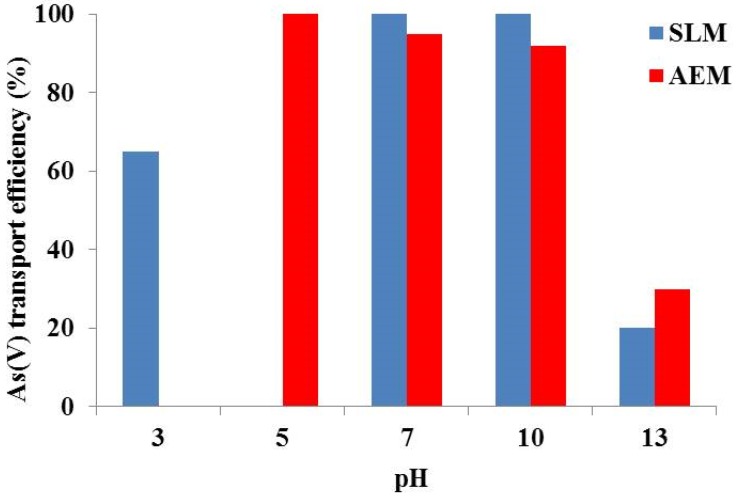
Influence of pH on As(V) transport efficiency after 24 h using SLM and AEM systems. (Feed phase: [As(V)] = 10 mg L^–1^; SLM: [Aliquat 336] = 0.5 M in dodecane and 4% dodecanol. Stripping phase: [NaCl] = 0.1 M [54].

Mafu *et al.* [52] used Aliquat 336 and sodium hydroxide as the extractant and stripping phase, respectively, optimizing the operational conditions and thus obtaining a 78% arsenic removal from real wastewater. In addition, Guell *et al.* [53] reported on As(V) and As(III) removal using a Aliquat 336 in a SLM system. As organic solvent a dodecane/dodecanole mixture at pH 13 was used. The receiving phase consisted of a 0.1 M HCl solution. The proposed system allowed for As(V) separation from As(III), which was driven by different kinetics, and the removal of the As(V) ions from real aqueous solutions. In particular, using a wastewater model prepared with ultrapure water, a complete recovery of arsenic was registered after 6 h. By contrast, when real tap water was used, a 44% recovery was obtained, demonstrating that the presence of other chemical compounds could negatively influence the As(V) recovery. Successively, a comparison between two membrane-based systems was analyzed [54] using natural water and operating at neutral pH values. The performance of two anion-exchange membranes (AEMs) was compared to that of the SLM previously described [54] and also in this case the effect of pH on the arsenic transport was evaluated. The authors reported the best results when pH 7 was used (Figure 6). Furthermore, a study on the As(III) ions transport under the optimal operational conditions, registered for As(V) at pH 7, was presented. The obtained data revealed that when an AEM system was used, As(III) ion transport was observed, although the process occurred at a lower rate in comparison to that obtained for As(V). On the contrary, the SLM system led to worsen results, due to the formation, at pH 7, of the undissociated H_3_AsO_3_ form, which could not be extracted by the Aliquat 336 anion exchanger. In view of the reported results, it can be assumed that the SLM allows for efficient As(III) and As(V) separation.

**Figure 7:**
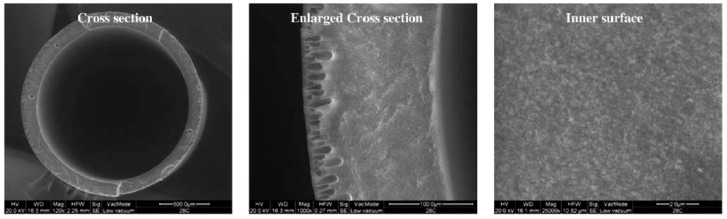
Morphology of the prepared PVDF hollow fiber membrane with 190 μm thickness. Magnification: cross section 120×; enlarged cross section 1000×; inner surface 25000× [28]. Reproduced with permission.

Bey *et al.* [28] prepared microporous hydrophobic polyvinilidene fluoride (PVDF) hollow fibers *via* by the dry/wet spinning technique. These fibers were experimented upon in laboratory-scaled membrane contactor modules for the extraction of As(V) from dilute aqueous solutions. Aliquat 336 in kerosene was chosen to extract the As(V) ions. In order to obtain fibers with 0.2 μm pore size and 80% porosity, the effect of spinning parameters such as bore fluid composition and flow rate was studied. Particularly promising was the membrane with the lowest thickness (190 μm) due to its low resistance and, at the same time, the high contact between the feed and recovery solution (Figure 7).

The support showed an asymmetric structure, composed of a sponge-like layer and finger-like macrovoids in the inner and the outer surface, respectively.

Experiments on arsenic removal were carried out using the optimal operational conditions previously identified (aqueous and organic phase flow rate of 0.47 mL/s and 1.4 mL/s, respectively). In an 8-h run, more than 75% of the arsenic was removed, which corresponded to an arsenic concentration reduction from 118 ppm to 25 ppm. These encouraging results could contribute to the development and application of an efficient hollow fiber contactor at industrial scale for the removal of arsenic from water.

Cyanex 921(trioctylphosphine oxide) and Na_2_SO_4_ were employed by Martinez Perez *et al.* [55] as highly efficient extractant and stripping compounds, respectively, for As(V) ion uptake from sulfuric acid solutions. Flat commercial PVDF membrane was used as support for the used LM system. The effect of different parame**t**ers such as stripping solution nature, stirring rate and extract content was examined. In particular, the study of the influence of the acid sulfuric concentration on As(V) removal revealed that by using a concentration of 1.6 M, 90% As(V) extraction was observed (Table 1).

**Table 1:** As(V) extraction as a function of sulfuric acid concentration, reported by Martinez Perez *et al.* [55].

Sulfuric acid concentration (mol/L)	% As(V) Extraction
~0.25	20
~0.6	45
~1.0	85
~1.6	90
~2.0	65
~2.4	30

Interestingly, the arsenic process in the SLM system occurred in five steps. Firstly, the metal ions propagated from the feed to the non-stirred boundary layer of the membrane. Then, the formation of the complex As(V)-extractant took place at the feed solution–SLM interface. The As(V)-Cyanex 921 complex diffused through the SLM, As(V) was stripped at the SLM–stripping solution interface, and, finally, arsenic ions spread in the non-stirred boundary layer at the strip membrane interface. The experimental system showed good selectivity in the arsenic removal, thus opening new prospects for wastewater purification from As(V) ions.

Tsai *et al.* [56] reported the separation of As(III) and Ga(III) using a SLM impregnated with ethylhexylphosphonic acid mono 2-ethylhexyl ester. They performed laboratory and pilot tests, registering a good separation between the two ion species in both systems. A hydrophobic polytetrafluoroethylene (PTFE) membrane showed the best performance compared to other tested membranes.

In the patent of Takigawa *et al.* [57] a SLM system based on the use of a polybenzimidazole membrane with extractant mixture in its pores was presented as an efficient system for the separation of hazardous metal ions such as arsenic, cadmium, antimony, palladium, *etc*. Thioxine was proposed as a complexing agent for some of the preselected toxic chemicals ions, including arsenic.

ELM systems for arsenic removal and recovery from water have not been extensively investigated so far. Among the few works in the literature, that of Mousavi *et al.* [58] was based on experimentation with a laboratory-scale ELM process for As(V) removal. The process proceeded with the emulsion preparation, the solute transfer, and the emulsion separation from the feed. An aqueous solution of sodium sulfate was used as internal phase, Span 80 (sorbitan mono-oleate) as emulsifier, Cyanex 921 as carrier, paraffin as membrane solvent, and water containing 50 ppm As(V) and sulfuric acid at different concentrations as the feed phase. Using this system, nine different experiments, carried out using different concentrations of sulfuric acid, reagent, carrier, and stirring speed, were carried out. The extraction rate was registered every three minutes, and that of the first extraction was the highest. In fact, after the initial phase, an increase of the arsenic content in the water was observed, probably due to the breakup of the inner droplets. An extraction of about 40% was obtained when 1.5 M sulfuric acid, 0.1 M carrier, and 1.5 M reagent concentrations were used, operating at a stirring speed of 500 rpm.

Li *et al.* [59] described the separation process of As(III) and As(V) using an ELM system formed by monosuccimide L113A, liquid paraffin, and kerosene as surfactant, stabilizer, and solvent, respectively. An HCl solution was used as the external phase, while KOH solution was chosen as the internal one. The permeation of As(III) through the membrane into the internal phase was promoted by the AsCl_3_ formation [60], where it was hydrolyzed to AsO3^3–^. By contrast, As(V) could not pass through the membrane, probably due to the [AsCl_4_]^+^[AsCl_6_]^–^ formation. The best results on the recovery of As(III), corresponding to a recovery above 95%, were obtained by adopting the following operational conditions:

- Internal phase: 2 M KOH;
- Membrane phase: 6% (v/v) L113A and 4% (v/v) liquid paraffin;
- External phase: 4 mg/L As(III) and 7 M HCl;
- Phase ratio: 2 oil/ 3 internal and 1 emulsion/5 water;
- Agitation speed: 400 rotations/minute;
- Extraction time: 10 minutes

Working under the abovementioned operational conditions, only 0.8% As(V) was extracted, but after the use of 0.6% KI, a quantitative reduction of As(V) into the As(III) form was observed, allowing for the determination of the total arsenic content.

In the ELM separation process, emulsion formation plays a crucial role in arsenic removal. To obtain a stable emulsion, the formation of small internal droplets is desired; their breakup can be efficiently decreased by using surfactant compounds. Treatment with these molecules leads to a reduced droplet dimension and an improved emulsion stability. The chemical structure of the selected surfactant and that of the two ELM phases strongly affects the degree of surfactant adsorption. Considering this, Kiani *et al.* [61] presented a work on the ultrasound-assisted preparation of stable water in an oil emulsion. The influence of different parameters, such as the surfactant type on droplet dimension, extraction amount, emulsifier content, and sonication time, were investigated. Sulfuric acid was used as the feed phase, with sodium sulfate as the internal phase reagent, Cyanex 921 as carrier, Span 80 (sorbitan mono-oletae) as lipophilic emulsifier, Tween 20 (polyoxyethylene (20) sorbitan mono-laurate) as hydrophilic surfactant, and liquid paraffin as membrane solvent; the removal of As(III) oxide—which was oxidized to As(V) oxide—was the target.

Several experiments were conducted by varying the Tween 20 concentration in the emulsifier mixture (0–30 wt%), the emulsifier content (2–7 vol%), and the sonication time (0–7 min). Experimental data showed that the ultrasound waves did not firmly lead to a reduction in the droplet size, probably due to the paraffin viscosity, which promotes the cavitation threshold and produces a decreased cavitation activity [62]. In the first 4 min of the experiment, carried out with a Span 80 concentration of 5 vol%, the increase in sonication time led to an increase in the emulsion temperature, causing a concomitant decrease of the interfacial tension and viscosity and, consequently, improving the emulsification process. After 4 min, the sonication time had an opposite effect, due to the re-coalescence of emulsion droplets (Table 2).

**Table 2:** Influence of sonication time on emulsion droplet size using 5 vol % Span 80 as emulsifier [61].

Sonication time (minutes)	Mean droplet diameter (μm)
1	2.3
3	1.9
4	1.7
7	3.7

The effect of Span 80 concentration on the arsenic extraction efficiency shown when using 4 vol % led to the best results because of the decrease in the internal droplet dimension, which resulted in increased mass transfer (Table 3).

**Table 3:** Influence of Span 80 content on As(V) in the feed phase during the extraction operation [61].

Span 80 concentration (vol %)	Extraction time (minutes)	As(V) in the feed phase (ppm)
2	3	37
2	6	45
2	9	47
4	3	15
4	6	19
4	9	21
5	3	20
5	6	26
5	9	34
7	3	34
7	6	36
7	9	42

In order to further improve the emulsion stability [63], a mixture of Span 80 and Tween 20 emulsifier was used. The registered data highlighted that emulsifier blends with a Tween 20 concentration of 11 vol % provided an enhanced emulsion stability in the time, decreasing the droplet size and, at the same time, enhancing the As(V) extraction amount.

Although LMs represent a valid alternative to the traditional separation methods, there were no significant developments at a large scale due to the insufficient membrane stability. This drawback was particularly remarkable for the SLMs and seemed to be related to the loss of the solvent and/or the carrier from the membrane during the time, thus affecting both the selectivity and the flux [64]. The membrane performance generally worsened over time, varying from 1 h to several months [65,66]. Among the causes that are considered responsible for the degradation, the progressive penetration of the aqueous phase in the pores of the membrane support [67,68], the mutual solubility of compounds from the aqueous to the LM phase [69], and the formation of emulsion in the LM phase [70] are the most frequent mechanisms. Moreover, in other studies many authors also attributed the poor stability to a difference in pressure over the membrane [71] and to the carrier complex precipitation at the membrane surface with the consequent blockage of membrane pores [72]. The stability control during that time also presents a problem. In fact, the existing methods that provide the measurements of both the membrane liquid loss and the water content that penetrate into the pores, are generally destructive and necessitate the use of a new SLM. To improve the LM stability, Molinari *et al.* [73] and Chiarizia *et al.* [74] proposed a nonstop support reimpregnation with LM phase, but the drawback of contaminated feed and/or strip solution(s) still persists in the system. Another feasible operation consists of plasma polymerization surface coating, which could stabilize the SLM [75] but at the same time could reduce the pore size in the membrane surface and decrease the membrane permeability. Also, interfacial polymerization and physical deposition [76,77] could create a barrier layer in the membrane surface, preventing the emulsification of the membrane liquid in the aqueous phase. By contrast, the presence of a polymerized film could reduce the membrane permeability; moreover, its poor adhesion on the SLM substrate leads to an unstable system.

## 3. Conclusions

Arsenic occurrence in water has been reported in many countries in the world and represents a concrete, critical problem for thousands of people who are exposed to this toxic (carcinogenic and mutagenic) metal primarily through water, the environment, and food.

The development of new, efficient technologies for arsenic removal is a challenging task, particularly in view of more and more severe environmental constraints. Furthermore, the need to reduce the costs of water purification encourages the search for tailored innovative methodologies.

The use of LMs is one of the most promising solutions. In particular, as they combine extraction with subsequent stripping in a single step, SLM and ELM represent the two most attractive LM configurations.

The efficiency of these processes has been investigated by several authors, who showed that the choice of extractant/surfactant is crucial in the optimization process. Several organic compounds have been tested for the SLM configuration, such as dibutyl butylphosphonate (DBBP), tributyl phosphate (TBP), Cyanex 923 (a mixture of phosphine oxides), Cyanex 301 (bis(2,4,4-trimethylpentyl), dithiophosphinic acid), tri-n-octylamine triocthyamine (TOA), methyltrioctylammonium chloride (Aliquat 336), *etc.* Among all of these, Aliquat 336 appears as a highly recommended extractant compound. It has attracted great attention, especially for HFSLM systems, due to its ability to form complexes with metal ions, thus promoting the movements of polluting compounds from the feed phase to the receiving phase. The possibility to remove and recover arsenic and mercury species from real water deriving from gas separation plants has been successfully obtained using HFSLMs, the use of which has brought below the regulatory limits the arsenic concentrations in the purified water.

Even though ELMs have not been as extensively studied as EMLs yet, they have also shown good results for arsenic removal, especially when Span 80 (sorbitan mono-oleate) or its blends with other compounds were employed as an organic surfactant.

In conclusion, in view of what is currently reported in the literature, it can be highlighted that LMs represent an innovative methodology to successfully perform arsenic removal from water, offering up the possibility of obtaining high selectivity and feasibility combined with an environmental friendly process. However, further investigation is still needed at this time to improve the various methods and overcome crucial issues that nowadays still prevent us from scaling up LM-based processes, such as, for instance, the poor stability of LMs over time.
